# Supplementary material for: Multi-Omics Analysis of Diabetic Heart Disease in the db/db Model Reveals Potential Targets for Treatment by a Longevity-Associated Gene
Source: Cells. 2020 May 21;9(5):1283. doi: 10.3390/cells9051283 (PMC7290798; doi:10.3390/cells9051283)
Supplement: Supplementary file 1 [file cells-09-01283-s001.pdf]

Supplementary Materials

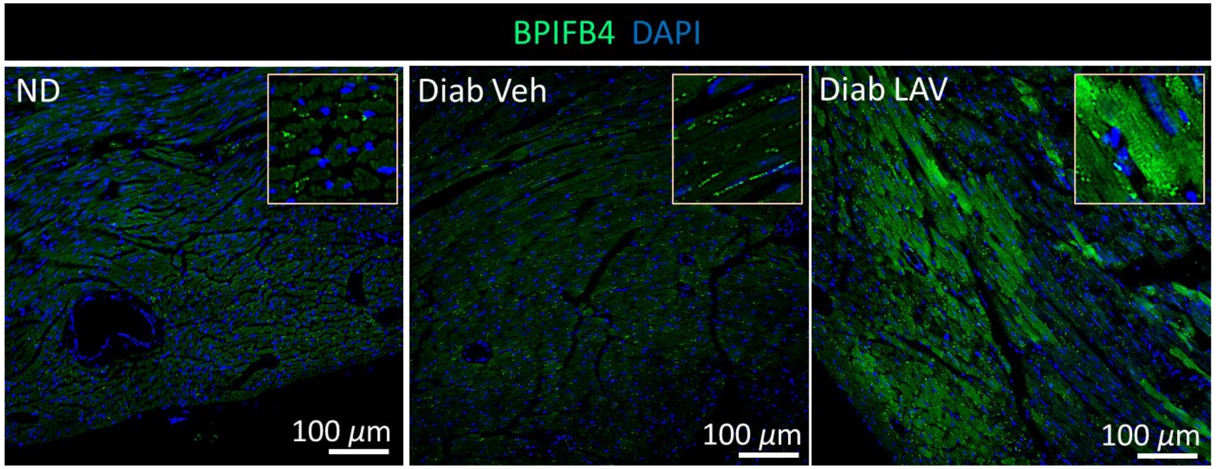

Supplementary Figure S1. Expression of BPIFB4 in murine hearts. Representative immunohistochemistry images showing the expression of BPIFB4 in the left ventricle of non-diabetic mice (ND) and diabetic mice (Diab) given vehicle or *LAV-BPIFB4.* BPIFB4 is shown in green, nuclei are identified by the blue fluorescence of DAPI. Scale bars: 1 mm and 100 μm.

Supplementary Table S1 – List of PCR primers.

Target gene		Primer Sequence	BI Accession number / Reference
Actb	Forward	AAGTCCCTCACCCTCCCAA	[1]
	Reverse	AAGCAATGCTGTCACCTTC	
Cpt1b	Forward	TCTAGGCAATGCCGTTCAC	[2]
	Reverse	GAGCACATGGGCACCATAC	
Acadm (Mcad)	Forward	GGAAATGATCAACAAAAAAAGAAGTATTT	[2]
	Reverse	GCCGCCACATCAGA	
Acot1	Forward	TGATGGTTTGGAGGTTGGGG	NM_012006.2
	Reverse	TGAAACTCCATTCCCAGCCC	
Hmgcs2	Forward	GGTGTCCCGTCTAATGGAGA	NM_008256.4
	Reverse	ACACCCAGGATTCACAGAGG	
Cs	Forward	CAAGCAGCAACATGGGAAGA	[2]
	Reverse	GTCAGGATCAAGAACCGAAGTCT	
Ucp3	Forward	GCCATTGTCAACTGTGCTGA	NM_009464.3
	Reverse	TCCTGAGCCACCATCTTCAG	
Atp5b	Forward	CGTGAGGGCAATGATTTATACCAT	[2]
	Reverse	TCCTGGTCTCTGAAGTATTCAGCAA	
Pdk4	Forward	CCGCTGTCCATGAAGCA	[2]

	Reverse	GCAGAAAAGCAAAGGACGTT	
Tomm20	Forward	AGAGTCCTATGCAGCCCAGA	NM_024214.2
	Reverse	CAAAGCCCCACATCTGTCCT	
Drp1	Forward	GCCTCAGATCGTCGTAGTGG	NM_152816.3
	Reverse	TTCCATGTGGCAGGGTCATT	
Mfn2	Forward	GGGAAGGTGAAGAAGCTTGGA	NM_001285920.1
	Reverse	ACAACTGGAACAGAGGAGAAGTT	
Ppargc1a (Pgc1α)	Forward	CGGAAATCATATCCAACCAG	[2]
	Reverse	TGAGAACCGCTAGCAAGTTTG	
Tfam	Forward	AGGCTTGGAAAAATCTGTCTC	[2]
	Reverse	TGCTCTTCCCAAGACTTCATT	
Bcl2l1	Forward	TGCCCCAGAGCTGTTAATGA	NM_001289716.1
	Reverse	TCTTCTTCCTGCCCTTCCTG	
Vwa3a	Forward	CCTTGTGGCCTCTCTGAAGA	NM_177697.3
	Reverse	CCGTCCCTTCTTTCTCAGGT	
Tlr1	Forward	CACCCCTACAGAAACGTCCT	NM_030682.2
	Reverse	TCCTTGGGCACTCTGGTAAG	
Fam107a	Forward	TCCCCACCCATTCTTCTTCC	NM_183187.3
	Reverse	GGGGAGTGAGAACTTCTGCT	
Gemin4	Forward	CCTCTTTTGGCCATGTTGCT	NM_177367.3
	Reverse	TGGTATGGGTTCTGGGTGTC	

Map3k6	Forward	GGAGTCCCAGCAGAAACCTC	NM_016693.5
	Reverse	GGCACTCACGTTCCTTCTCA	
Ky	Forward	AGTCCCCTCTGCCTCAATCT	NM_024291.3
	Reverse	ACCTCCGCTTTCCCATTCAG	
Rgcc	Forward	GTCACTCCTCGGAAAGCCAA	NM_025427.2
	Reverse	AGCTTCACTCTCCGAACTGC	
Timp4	Forward	CTTCCCTGTCTCCCAAACCC	NM_080639.3
	Reverse	GTCAGCCCACCTACCATAGC	

References

1. Ferland-McCollough, D.; Maselli, D.; Spinetti, G.; Sambataro, M.; Sullivan, N.; Blom, A.; Madeddu, P. MCP-1 Feedback Loop Between Adipocytes and Mesenchymal Stromal Cells Causes Fat Accumulation and Contributes to Hematopoietic Stem Cell Rarefaction in the Bone Marrow of Patients With Diabetes. *Diabetes* **2018**, *67*, 1380, doi:10.2337/db18-0044.

2. Duncan, J.G.; Fong, J.L.; Medeiros, D.M.; Finck, B.N.; Kelly, D.P. Insulin-Resistant Heart Exhibits a Mitochondrial Biogenic Response Driven by the Peroxisome Proliferator-Activated Receptor-α/PGC-1α Gene Regulatory Pathway. *Circulation* **2007**, *115*, 909-917, doi:10.1161/circulationaha.106.662296.

Supplementary Table S2: List of western blotting antibodies

Target protein	Supplier	Catalogue number	Dilution
**CPT1B**	Abcam	Ab134988	1:1000
**TOMM20**	Abcam	Ab186735	1:2000
**ATPB**	Abcam	Ab14730	1:2000
**UCP3**	Abcam	Ab3477	1:500
**HMGCS2**	Abcam	Ab137043	1:1000
**PDK4**	ThermoFisher	PA5-22983	1:500
**MCAD**	Abcam	Ab92461	1:10000
**CIT SYN**	Cell Signaling Technology	14309	1:1000
**BPIFB4**	GeneTex	GTX51455	1:1000
**TFAM**	Abcam	Ab131607	1:2000
**PGC1α**	Abcam	Ab191838	1:1000
**MFN2**	Cell Signaling Technology	9482	1:1000
**DRP1**	Cell Signaling Technology	5391	1:1000
**ACOT1**	Abcam	Ab133948	1:1000
**β-Tubulin**	Cell Signaling Technology, rabbit or mouse mAb	86298/3700	1:1000/3000

Supplementary Table S3. Cardiac metabolites modulated by *LAV-BPIFB4* compared with *GFP* control

		GFP vs. LAV in Diabetes		
**Metabolite class**	**Metabolites**	***t*-test**	**Fold change**	**Note**
Acyl carnitine	2-Methylbutyroylcarnitine and/or Isovalerylcarnitine and/or Pivaloylcarnitine and/or Valerylcarnitine	0.04093	0.22	Higher in LAV
Acyl carnitine	Fumarycarnitine	0.00048	0.22	Higher in LAV
Amino sugar and nucleotide sugar metabolism	UDP-D-apiose and/or UDP-D-Xylose and/or UDP-L-arabinose	0.04776	0.05	Higher in LAV
Amino sugar and nucleotide sugar metabolism	GDP-glucose and/or Guanosine diphosphate mannose	0.00650	0.19	Higher in LAV
Amino sugar and nucleotide sugar metabolism	UDP-N-acetyl-D-mannosamine and/or Uridine diphosphate-N-acetylgalactosamine and/or Uridine diphosphate-N-acetylglucosamine	0.01805	0.21	Higher in LAV
Amino sugar and nucleotide sugar metabolism	Glucuronic acid 1-phosphate	0.02597	0.25	Higher in LAV
Aromatic metabolite	Hydroxybenzeneacetonitrile and/or Indoxyl	0.04220	0.05	Higher in LAV
Aromatic metabolite	2,6-Dimethoxy-1,4-benzoquinone and/or 3-Hydroxymandelic acid and/or 3,4-Dihydroxybenzeneacetic acid and/or Homogentisic acid and/or p-Hydroxymandelic acid	0.04519	0.08	Higher in LAV
Carbohydrate metabolism	2-deoxy-ribonic acid and/or 2-Deoxyribonic acid and/or Arabinofuranose and/or Arabinose and/or Beta-D-ribopyranose and/or D-Apiose and/or D-Ribose and/or D-Ribulose and/or D-Xylose and/or D-Xylulose and/or L-Arabinose and/or L-Ribulose	0.03655	0.23	Higher in LAV
Cardiolipin	CL (74:6) and/or CL (72:5)	0.04512	0.14	Higher in LAV
Cardiolipin	CL (78:14)	0.04456	0.15	Higher in LAV
Cardiolipin	CL (74:5)	0.04286	0.22	Higher in LAV
CoA metabolism	Pantothenol	0.00045	0.44	Higher in LAV
Diacylglyceride	DG(35:2)	0.02291	0.33	Higher in LAV
Folate metabolism	6-Methyltetrahydropterin	0.04690	0.16	Higher in LAV
Glycerophospholipid (mixed classes)	PE(38:7) and/or PC(33:4) and/or PE(P-16:0/20:4(12OH(S))) and/or PE(P-16:0/20:4(15OH(S))) and/or PE(P-16:0/20:4(5OH(S))) and/or PE(36:4)	0.01245	0.05	Higher in LAV

Glycerophospholipid (mixed classes)	PC(15:0/P-18:0) and/or PC(O-16:0/17:1) and/or PC(O-18:0/15:1) and/or PC(P-16:0/17:0) and/or PC(P-18:0/15:0) and/or PC(P-20:0/13:0) and/or PE(18:0/P-18:0) and/or PE(20:0/P-16:0) and/or PE(dm18:0/18:0) and/or PE(O-16:0/20:1) and/or PE(O-18:0/18:1) and/or PE(O-20:0/16:1) and/or PE(P-16:0/20:0) and/or PE(P-20:0/16:0) and/or PA(O-16:0/22:2) and/or PA(O-18:0/20:2) and/or PA(O-20:0/18:2) and/or PA(P-16:0/22:1) and/or PA(P-18:0/20:1) and/or PA(P-20:0/18:1) and/or Cer(d18:1/24:1)	0.02301	0.10	Higher in LAV
Glycerophospholipid (mixed classes)	PC(35:6) and/or PE(P-16:0/22:6(14OH)) and/or PE(38:6) and/or PC(33:3) and/or PE(36:3) and/or PA(40:7) and/or PC(31:0) and/or PE-NMe2(32:0) and/or PE(34:0) and/or PC(O-16:0/15:0) and/or PC(O-18:0/13:0) and/or PE(O-16:0/18:0) and/or PE(O-18:0/16:0) and/or PE(O-20:0/14:0)	0.02636	0.13	Higher in LAV
Glycerophospholipid (mixed classes)	PS(43:1) and/or PC(P-20:0/21:0) and/or PC(22:0/P-18:0) and/or PC(24:0/P-16:0) and/or PC(O-18:0/22:1) and/or PC(o-18:1/22:0) and/or PC(O-20:0/20:1) and/or PC(P-16:0/24:0) and/or PC(P-18:0/22:0) and/or PC(P-20:0/20:0) and/or PC(40:2) and/or PE(43:2)	0.01886	0.14	Higher in LAV
Glycerophospholipid (mixed classes)	PC(32:2) and/or PE(35:2) and/or PA(37:3)	0.0066	0.15	Higher in LAV
Glycerophospholipid (mixed classes)	PG(O-16:0/21:0) and/or PG(O-18:0/19:0) and/or PG(O-20:0/17:0) and/or PS(37:3) and/or PA(41:1) and/or PG(O-16:0/19:1) and/or PG(O-18:0/17:1) and/or PG(O-20:0/15:1) and/or PG(P-16:0/19:0) and/or PG(P-18:0/17:0) and/or PG(P-20:0/15:0) and/or PA(O-18:0/21:0) and/or PA(O-20:0/19:0)	0.00317	0.17	Higher in LAV
Glycerophospholipid (mixed classes)	PE(42:10) and/or PS(O-18:0/20:5) and/or PS(P-16:0/22:4) and/or PS(P-18:0/20:4) and/or PS(P-20:0/18:4) and/or PI(33:4) and/or PC(37:7) and/or PE(40:7) and/or PS(35:1) and/or PE(20:4/P-18:1) and/or PE(20:5/P-18:0) and/or PE(22:5/P-16:0) and/or PE(O-16:0/22:6) and/or PE(P-16:0/22:5) and/or PE(P-18:0/20:5) and/or PE(P-18:1/20:4) and/or PC(14:0/P-18:0) and/or PC(16:0/P-16:0) and/or PC(O-14:0/18:1) and/or PC(o-16:0/16:1) and/or PC(O-18:0/14:1) and/or PC(P-16:0/16:0) and/or PC(P-18:0/14:0) and/or PC(P-20:0/12:0) and/or PE(O-16:0/19:1) and/or PE(O-18:0/17:1) and/or PE(O-20:0/15:1) and/or PE(P-16:0/19:0) and/or PE(P-18:0/17:0) and/or PE(P-20:0/15:0)	0.02375	0.21	Higher in LAV
Glycerophospholipid (PC)	PC(46:3) and/or PC(44:0)	0.0051	0.15	Higher in LAV
Glycerophospholipid (PC/PE)	PC(18:3/P-18:1) and/or PC(18:4/dm18:0) and/or PC(18:4/P-18:0) and/or PC(20:4/P-16:0) and/or PC(O-16:0/20:5) and/or PC(o-16:1/20:4) and/or	0.01062	0.13	Higher in LAV

	PC(P-16:0/20:4) and/or PC(P-18:0/18:4) and/or PC(P-18:1/18:3) and/or PC(16:0/P-18:1) and/or PC(16:1/P-18:0) and/or PC(18:1/P-16:0) and/or PC(O-16:0/18:2) and/or PC(P-16:0/18:1) and/or PC(P-18:0/16:1) and/or PC(P-18:1/16:0) and/or PC(P-20:0/14:1) and/or PE(O-20:0/17:2) and/or PE(P-18:0/19:1) and/or PE(P-20:0/17:1)			
Glycerophospholipid (PC/PE)	PC(29:2) and/or PE(32:2) and/or PA(34:3)	0.02603	0.14	Higher in LAV
Glycerophospholipid (PC/PE)	PC(33:3) and/or PE(36:3) and/or PC(31:0) and/or PE-NMe2(32:0) and/or PE(34:0)	0.03221	0.15	Higher in LAV
Glycerophospholipid (PC/PE)	PC(29:2) and/or PE(32:2) and/or PA(34:3)	0.03835	0.22	Higher in LAV
Glycerophospholipid (PE)	PE(38:7)	0.02930	0.09	Higher in LAV
Glycerophospholipid (PS)	PS(39:5)	0.04740	0.24	Higher in LAV
Glycerophospholipid metabolism	Glycerophosphocholine	0.00226	0.26	Higher in LAV
Mixed classes	Ethylphosphate and/or 2-Furoic acid and/or 3-Furoic acid	0.04199	0.02	Higher in LAV
Mixed classes	3,4-Dihydroxyphenylglycol O-sulfate and/or Hydroxy-octadienoic acid	0.02088	0.06	Higher in LAV
Mixed classes	PC(34:5) and/or PE(37:5) and/or PC(32:2) and/or PE(35:2) and/or PA(39:6) and/or GlcCer(d15:2/18:0)	0.00286	0.08	Higher in LAV
Mixed classes	ADP-glucose and/or ADP-Mannose and/or GDP-D-Rhamnose and/or GDP-L-fucose and/or 10-Formyltetrahydrofolate and/or N5-Formyl-THF and/or Pteroyl-D-glutamic acid	0.00089	0.09	Higher in LAV
Mixed classes	N2-Succinyl-L-glutamic acid 5-semialdehyde and/or 6-Dimethylaminopurine	0.03966	0.13	Higher in LAV
Mixed classes	Homocystine and/or Mannitol 1-phosphate and/or Sorbitol-6-phosphate and/or 5-Acetylamino-6-formylamino-3-methyluracil	0.04503	0.13	Higher in LAV
Mixed classes	PC(P-16:0/13:0) and/or PE(14:0/P-18:0) and/or PE(16:0/P-16:0) and/or PE(O-16:0/16:1) and/or PE(O-18:0/14:1) and/or PE(P-16:0e/16:0) and/or PE(P-18:0/14:0) and/or PE(P-20:0/12:0) and/or PA(O-16:0/18:2) and/or PA(P-16:0/18:1) and/or PA(P-18:0/16:1) and/or PA(P-20:0/14:1) and/or Cer(d14:1/24:1) and/or Cer(d14:2/24:0) and/or Cer(d16:1/22:1) and/or Cer(d16:2/22:0) and/or Cer(d18:2/20:0)	0.01268	0.17	Higher in LAV
Mixed classes	PC(36:3) and/or PE(39:3) and/or PC(34:0) and/or PE-NMe(36:0) and/or PE(37:0) and/or PA(41:4) and/or Cer(d18:0/24:0(2OH)) and/or	0.01847	0.18	Higher in LAV

	Cer(d18:0/h24:0) and/or Cer(d20:0/22:0(2OH)) and/or Cer(t18:0/24:0) and/or Cer(t20:0/22:0)			
Mixed classes	N-Acetylneuraminic acid and/or Glutamyl-Tyrosine and/or Tyrosyl-Glutamate	0.03273	0.25	Higher in LAV
Mixed classes	Pantothenoyl-L-cysteine and/or Octadecenetriynoic acid and/or Estrone and/or 5-Amino-6-ribitylamino uracil and/or L-gamma-glutamyl-L-isoleucine and/or L-gamma-glutamyl-L-leucine	0.02269	0.26	Higher in LAV
Mixed classes	2-Phosphoglyceric acid and/or 3-Phosphoglyceric acid and/or Acetylphosphate	0.03192	0.28	Higher in LAV
Mixed classes	PE-NMe2(24:0) and/or PE (26:0) and/or PI(20:0) and/or CerP(d18:1/12:0)	0.00223	0.31	Higher in LAV
Mixed classes	10,11-dihydro-20-trihydroxy-leukotriene B4 and/or Oleandolide and/or 3-Oxo-4,6-choladienoic acid and/or -8,18-propano-retinal and/or 1alpha,17alpha,21-trihydroxy-20-oxo-22,23,24,25,26,27-hexanorcholecalciferol and/or 17a,21-Dihydroxy-5b-pregnane-3,11,20-trione and/or 18-Hydroxycorticosterone and/or 4,5alpha-Dihydrocortisone	0.02830	0.31	Higher in LAV
Mixed classes	11-deoxy-11-methylene-15-keto-PGD2 and/or 11b,21-Dihydroxy-5b-pregnane-3,20-dione and/or 17a,21-Dihydroxypreg-nenolone and/or 3a,21-Dihydroxy-5b-pregnane-11,20-dione and/or 3b,15b,17a-Trihydroxy-pregnenone and/or 3b,17a,21-Trihydroxypregnenone and/or 3beta,15beta,17alpha-Trihydroxy-pregnenone and/or 7'-Carboxy-alpha-chromanol	0.02909	3.72	Higher in GFP
Multiple classes	Lactic acid	0.01014	0.06	Higher in LAV
Multiple classes	Pyruvic acid	0.00775	0.25	Higher in LAV
Multiple classes	Glycine	0.00141	0.39	Higher in LAV
Nicotinate and nicotinamide metabolism	Iminoaspartic acid	0.01642	0.30	Higher in LAV
Other class	1-(sn-Glycero-3-phospho)-1D-myo-inositol	0.00804	0.27	Higher in LAV
Other class	Pyrrolidine	0.01396	0.39	Higher in LAV
Pentose phosphate pathway	2-Keto-3-deoxy-6-phosphogluconic acid and/or 6-Phosphonoglucono-D-lactone	0.03749	0.23	Higher in LAV
Peptide	S-Formylglutathione and/or Cysteinyl-Arginine and/or Arginyl-Serine and/or Glutamyl-Valine and/or Valyl-Glutamate	0.03463	0.13	Higher in LAV
Peptide	Cysteinyl-Gamma-glutamate and/or Cysteinyl-Glutamine	0.02723	0.28	Higher in LAV
Peptide	Glutamyl-Valine and/or Valyl-Glutamate	0.02138	7.54	Higher in GFP

Short chain organic acid	Citraconic acid and/or Glutaconic acid and/or Itaconic acid and/or Mesaconic acid	0.02905	0.12	Higher in LAV
Steroid and sterol metabolism	7-beta-estradiol 3-sulfate-17-(beta-D-glucuronide)	0.00943	0.11	Higher in LAV
Sterol ester	CE(22:1) and/or Sitosteryl ester(20:1) and/or Stigmasteryl ester(20:0)	0.01864	0.15	Higher in LAV
Terpenoid backbone metabolism	4-hydroxy-3-methylbut-2-en-1-yl trihydrogen diphosphate	0.03805	0.11	Higher in LAV
Thiol metabolite	2-Amino-5-(methylthio)pentanoic acid	0.01498	0.20	Higher in LAV
Triacylglyceride	TG(57:6)	0.01935	10.28	Higher in LAV
Triacylglyceride	TG(57:7)	0.01464	14.73	Higher in GFP
Tryptophan metabolism	1H-Indole-3-carboxaldehyde	0.04624	0.08	Higher in LAV

Supplementary Table S4 Cardiac metabolites differentially modulated in both the comparisons between ND *vs*. diabetes and *GFP vs. LAV*

		ND *vs. GFP*-treated diabetics		*GFP*- vs. *LAV*-treated diabetics		Notes
**Metabolite class**	**Metabolites**	***t*-test (*p* value)**	**Fold change**	***t*-test (*p* value)**	**Fold change**	
Other class	Pyrrolidine	0.01402	0.18	0.01396	0.39	higher in GFP vs. ND<br>higher in LAV vs. GFP
Glycerophospholipid (mixed classes)	PC(P-16:0/13:0) and/or PE(14:0/P-18:0) and/or PE(16:0/P-16:0) and/or PE(O-16:0/16:1) and/or PE(O-18:0/14:1) and/or PE(P-16:0e/16:0) and/or PE(P-18:0/14:0) and/or PE(P-20:0/12:0) and/or PA(O-16:0/18:2) and/or PA(P-16:0/18:1) and/or PA(P-18:0/16:1) and/or PA(P-20:0/14:1)	0.00007	0.58	0.01268	0.17	higher in GFP vs. ND<br>higher in LAV vs. GFP
Glycerophospholipid (mixed classes)	PC(34:5) and/or PE(37:5) and/or PC(32:2) and/or PE(35:2) and/or PA(39:6)	0.00035	0.40	0.00286	0.08	higher in GFP vs. ND<br>higher in LAV vs. GFP
Glycerophospholipid (mixed classes)	PG(O-16:0/21:0) and/or PG(O-18:0/19:0) and/or PG(O-20:0/17:0) and/or PS(37:3) and/or PA(41:1) and/or PG(O-16:0/19:1) and/or PG(O-18:0/17:1) and/or PG(O-20:0/15:1) and/or PG(P-16:0/19:0) and/or PG(P-18:0/17:0) and/or PG(P-20:0/15:0) and/or PA(O-18:0/21:0) and/or PA(O-20:0/19:0)	0.00195	0.29	0.00317	0.17	higher in GFP vs. ND<br>higher in LAV vs. GFP

Glycerophospholipid (mixed classes)	PS(43:1) and/or PC(P-20:0/21:0) and/or PC(22:0/P-18:0) and/or PC(24:0/P-16:0) and/or PC(O-18:0/22:1) and/or PC(o-18:1/22:0) and/or PC(O-20:0/20:1) and/or PC(P-16:0/24:0) and/or PC(P-18:0/22:0) and/or PC(P-20:0/20:0) and/or PC(40:2) and/or PE(43:2)	0.00021	2.14	0.01886	0.14	lower in GFP vs. ND higher in LAV vs. GFP
Glycerophospholipid (PS)	PS(39:5)	0.00701	0.37	0.04740	0.24	higher in GFP vs. ND higher in LAV vs. GFP
Glycerophospholipid (mixed classes)	PC(32:2) and/or PE(35:2) and/or PA(37:3)	0.00240	0.21	0.00661	0.15	higher in GFP vs. ND higher in LAV vs. GFP
Amino sugar and nucleotide sugar metabolism	UDP-D-apiose and/or UDP-D-Xylose and/or UDP-L-arabinose	0.00382	0.50	0.04776	0.05	higher in GFP vs. ND higher in LAV vs. GFP
Amino sugar and nucleotide sugar metabolism	ADP-glucose and/or ADP-Mannose and/or GDP-D-Rhamnose and/or GDP-L-fucose	0.00104	0.23	0.00089	0.09	higher in GFP vs. ND higher in LAV vs. GFP
Cardiolipin	CL (78:14)	0.00019	3.03	0.04460	0.15	lower in GFP vs. ND higher in LAV vs. GFP
Cardiolipin	CL (74:6) and/or CL (72:5)	0.00013	3.26	0.04512	0.14	lower in GFP vs. ND higher in LAV vs. GFP
Terpenoid backbone metabolism	4-hydroxy-3-methylbut-2-en-1-yl trihydrogen diphosphate	0.04063	0.37	0.03805	0.11	higher in GFP vs. ND higher in LAV vs. GFP

Supplementary Table S5: List of genes differentially expressed in hearts from vehicle-treated diabetic and non-diabetic mice.

GENE ID	GENE BIOTYPE	FDR STEP UP	FOLD CHANGE (DIAB *vs.* ND)	TOTAL COUNTS	*p*-VALUE	RATIO	LS MEAN DIAB	LS MEAN ND
Retnla+A25I1A2:A26 A2:A52	protein_coding	9.32E-03	-3.23	2.40E+01	1.08E-04	3.10E-01	1.26E+00	4.07E+00
H2-Aa	protein_coding	1.10E-02	-3.22	2.17E+02	1.51E-04	3.11E-01	1.18E+01	3.79E+01
H2-Ab1	protein_coding	9.48E-03	-3.07	1.05E+02	1.14E-04	3.26E-01	6.01E+00	1.84E+01
H2-Eb1	protein_coding	8.13E-03	-3.06	1.21E+02	7.14E-05	3.27E-01	7.01E+00	2.14E+01
H2-Ea-ps	unprocessed_ pseudogene	1.66E-02	-2.91	8.24E+01	3.57E-04	3.44E-01	4.89E+00	1.42E+01
Cd74	protein_coding	1.21E-02	-2.55	3.71E+02	1.83E-04	3.92E-01	2.42E+01	6.17E+01
Gm43565	lincRNA	3.09E-02	-2.45	9.42E+00	1.23E-03	4.08E-01	6.25E-01	1.53E+00
Cpxm2	protein_coding	1.85E-02	-2.39	2.07E+02	4.72E-04	4.18E-01	1.31E+01	3.15E+01
Cdk5r1	protein_coding	4.11E-02	-2.39	1.32E+01	2.22E-03	4.18E-01	9.19E-01	2.20E+00
Mgl2	protein_coding	1.53E-02	-2.34	2.03E+01	3.07E-04	4.27E-01	1.33E+00	3.11E+00
Foxo6os	antisense	5.85E-03	-2.27	4.01E+01	2.79E-05	4.41E-01	2.96E+00	6.72E+00
C7	protein_coding	1.36E-02	-2.18	4.03E+01	2.34E-04	4.60E-01	2.94E+00	6.39E+00
Adap1	protein_coding	8.54E-03	-2.17	1.70E+01	9.01E-05	4.60E-01	1.20E+00	2.61E+00
Oas2	protein_coding	4.45E-02	-2.15	7.29E+01	2.60E-03	4.65E-01	5.45E+00	1.17E+01
2310069G16Rik	lincRNA	2.56E-02	-2.14	8.57E+00	8.48E-04	4.67E-01	6.23E-01	1.33E+00
Scgb1c1	protein_coding	4.11E-02	-2.12	4.71E+01	2.22E-03	4.73E-01	3.40E+00	7.19E+00
Gm45018	lincRNA	2.03E-02	-2.09	7.23E+00	5.55E-04	4.78E-01	4.85E-01	1.01E+00
Kcnc1	protein_coding	3.57E-02	-2.05	1.38E+01	1.67E-03	4.88E-01	9.58E-01	1.96E+00
Gm11408	processed_ pseudogene	8.38E-03	-2.05	2.33E+03	8.43E-05	4.88E-01	1.53E+02	3.14E+02
AW112010	lincRNA	1.66E-02	-2.03	9.95E+01	3.55E-04	4.92E-01	7.48E+00	1.52E+01
Fam122b	protein_coding	5.69E-03	2.01	6.58E+01	2.50E-05	2.01E+00	8.01E+00	3.99E+00
Sbk2	protein_coding	2.58E-03	2.03	5.33E+01	2.20E-06	2.03E+00	6.74E+00	3.33E+00

Opn4	protein_coding	4.73E-02	2.05	9.27E+00	2.91E-03	2.05E+00	1.29E+00	6.30E-01
Lrrc52	protein_coding	3.09E-02	2.11	6.26E+00	1.25E-03	2.11E+00	6.06E-01	2.87E-01
Ncam1	protein_coding	2.07E-02	2.12	3.30E+01	5.91E-04	2.12E+00	4.11E+00	1.94E+00
8430408G22Rik	protein_coding	4.48E-03	2.15	3.86E+02	5.61E-06	2.15E+00	4.03E+01	1.87E+01
Tox	protein_coding	1.36E-02	2.16	7.79E+00	2.39E-04	2.16E+00	1.01E+00	4.68E-01
Gm35013	processed_ pseudogene	1.66E-02	2.18	5.58E+00	3.62E-04	2.18E+00	7.74E-01	3.55E-01
Hif3a	protein_coding	1.73E-02	2.18	2.79E+01	3.90E-04	2.18E+00	2.71E+00	1.24E+00
Gm10603	antisense	2.15E-02	2.21	1.40E+01	6.24E-04	2.21E+00	1.50E+00	6.78E-01
Acot1	protein_coding	4.48E-03	2.22	1.71E+02	6.97E-06	2.22E+00	1.61E+01	7.27E+00
Slc41a3	protein_coding	2.43E-02	2.25	2.03E+02	7.51E-04	2.25E+00	2.29E+01	1.02E+01
Gm12100	processed_transc ript	1.78E-02	2.26	9.06E+00	4.35E-04	2.26E+00	1.20E+00	5.33E-01
Cacng6	protein_coding	1.18E-02	2.27	8.67E+00	1.75E-04	2.27E+00	1.09E+00	4.80E-01
Pirt	protein_coding	2.63E-02	2.37	8.91E+01	8.92E-04	2.37E+00	1.18E+01	4.98E+00
Gm10032	protein_coding	3.10E-02	2.37	7.09E+00	1.27E-03	2.37E+00	7.80E-01	3.29E-01
Tmem56	protein_coding	1.78E-02	2.38	1.74E+01	4.25E-04	2.38E+00	2.43E+00	1.02E+00
Hmgcs2	protein_coding	5.85E-03	2.42	2.84E+02	2.83E-05	2.42E+00	2.06E+01	8.52E+00
Ly75	protein_coding	8.38E-03	2.44	1.14E+01	8.53E-05	2.44E+00	1.70E+00	6.99E-01
Pdk4	protein_coding	6.86E-03	2.44	9.97E+03	3.67E-05	2.44E+00	8.08E+02	3.31E+02
Spock2	protein_coding	9.64E-04	2.51	1.48E+02	6.87E-08	2.51E+00	1.81E+01	7.20E+00
Edn3	protein_coding	4.59E-02	2.53	4.72E+01	2.75E-03	2.53E+00	5.79E+00	2.29E+00
Pdzrn4	protein_coding	4.48E-03	2.58	1.16E+01	5.40E-06	2.58E+00	1.55E+00	6.03E-01
Sdk2	protein_coding	6.81E-03	2.59	1.91E+01	3.50E-05	2.59E+00	1.46E+00	5.65E-01
Agt	protein_coding	9.48E-03	2.67	3.41E+01	1.14E-04	2.67E+00	4.39E+00	1.65E+00
Snai3	protein_coding	1.07E-02	2.69	5.93E+01	1.42E-04	2.69E+00	8.06E+00	3.00E+00
Fam46b	protein_coding	1.13E-02	2.71	8.74E+00	1.63E-04	2.71E+00	9.68E-01	3.58E-01
Gm31659	lincRNA	1.23E-02	2.80	1.68E+01	1.92E-04	2.80E+00	2.24E+00	8.01E-01
Lcn2	protein_coding	2.58E-03	2.86	1.78E+01	1.88E-06	2.86E+00	2.59E+00	9.07E-01

Cyp2b10	protein_coding	1.11E-02	2.87	9.99E+00	1.54E-04	2.87E+00	9.10E-01	3.17E-01
Abca4	protein_coding	2.08E-03	2.90	4.84E+01	5.55E-07	2.90E+00	6.18E+00	2.14E+00
Scube1	protein_coding	2.08E-03	2.90	5.32E+01	8.21E-07	2.90E+00	6.43E+00	2.22E+00
Kcne1	protein_coding	1.39E-02	2.96	1.00E+02	2.49E-04	2.96E+00	1.29E+01	4.34E+00
Sfrp5	protein_coding	1.30E-02	3.06	9.94E+00	2.16E-04	3.06E+00	1.25E+00	4.09E-01
Aqp4	protein_coding	4.60E-03	3.15	5.42E+01	1.54E-05	3.15E+00	7.45E+00	2.37E+00
Angptl4	protein_coding	3.44E-02	3.56	1.42E+02	1.54E-03	3.56E+00	1.17E+01	3.28E+00
AI593442	protein_coding	1.52E-02	3.84	2.37E+01	3.04E-04	3.84E+00	3.08E+00	8.01E-01
Klk1b26	protein_coding	5.77E-03	4.38	1.36E+01	2.67E-05	4.38E+00	2.01E+00	4.60E-01
Snhg11	protein_coding	2.21E-02	5.51	1.66E+01	6.48E-04	5.51E+00	2.68E+00	4.87E-01
Kcnk1	protein_coding	1.25E-02	6.00	7.14E+00	2.00E-04	6.00E+00	9.98E-01	1.66E-01

Supplementary Table S6: Genes differentially expressed in hearts from *LAV-BPIFB4*-treated diabetic mice and non-diabetic mice.

GENE ID	GENE BIOTYPE	FDR STEP UP	FOLD CHANGE (DIAB *LAV* *vs.* ND)	TOTAL COUNTS	*p*-VALUE	RATIO	LS MEAN DIAB *LAV*	LS MEAN ND
Hcn1	protein_coding	3.86E-03	-8.16	1.18E+01	1.86E-04	1.23E-01	2.52E-01	2.05E+00
Hspa1a	protein_coding	1.23E-02	-7.52	2.96E+01	1.16E-03	1.33E-01	7.95E-01	5.98E+00
Nos1	protein_coding	4.60E-03	-7.16	1.48E+01	2.49E-04	1.40E-01	3.69E-01	2.64E+00
Mmp13	protein_coding	1.45E-03	-5.84	7.11E+00	3.17E-05	1.71E-01	2.10E-01	1.23E+00
Apol10b	protein_coding	2.12E-03	-5.07	5.03E+01	6.49E-05	1.97E-01	1.67E+00	8.47E+00
Hspa1b	protein_coding	3.59E-02	-4.90	2.61E+01	6.11E-03	2.04E-01	1.00E+00	4.92E+00
Foxo6os	antisense	3.59E-05	-4.67	4.01E+01	4.10E-08	2.14E-01	1.44E+00	6.72E+00
Tgtp1	protein_coding	4.94E-02	-4.61	1.16E+02	9.92E-03	2.17E-01	4.39E+00	2.03E+01
H2-Eb1	protein_coding	4.98E-04	-4.50	1.21E+02	4.30E-06	2.22E-01	4.76E+00	2.14E+01
Oas1a	protein_coding	1.07E-03	-4.24	1.62E+01	1.77E-05	2.36E-01	6.11E-01	2.59E+00
H2-Ab1	protein_coding	9.61E-04	-4.19	1.05E+02	1.46E-05	2.39E-01	4.40E+00	1.84E+01
Cdk5r1	protein_coding	1.63E-03	-4.05	1.32E+01	3.97E-05	2.47E-01	5.42E-01	2.20E+00
H2-Ea-ps	unprocessed_pseudogene	1.63E-03	-3.91	8.24E+01	3.97E-05	2.56E-01	3.64E+00	1.42E+01
Gm4951	protein_coding	2.39E-02	-3.86	1.14E+01	3.36E-03	2.59E-01	4.98E-01	1.92E+00
H2-Aa	protein_coding	2.14E-03	-3.74	2.17E+02	6.56E-05	2.68E-01	1.01E+01	3.79E+01
Ifi213	protein_coding	4.00E-03	-3.72	1.34E+01	1.99E-04	2.69E-01	5.80E-01	2.16E+00
Cd74	protein_coding	8.52E-04	-3.61	3.71E+02	1.06E-05	2.77E-01	1.71E+01	6.17E+01
Oas2	protein_coding	1.77E-03	-3.60	7.29E+01	4.57E-05	2.77E-01	3.25E+00	1.17E+01
Acta1	protein_coding	1.07E-02	-3.60	2.53E+03	9.41E-04	2.78E-01	1.17E+02	4.19E+02
Cxcl9	protein_coding	2.22E-02	-3.56	3.77E+01	3.02E-03	2.81E-01	1.65E+00	5.87E+00
Tlr1	protein_coding	2.20E-04	-3.44	8.09E+00	1.00E-06	2.91E-01	3.55E-01	1.22E+00
Isg15	protein_coding	7.81E-03	-3.43	2.02E+01	5.81E-04	2.91E-01	8.62E-01	2.96E+00
Ciita	protein_coding	6.49E-03	-3.43	1.04E+01	4.25E-04	2.92E-01	4.84E-01	1.66E+00

H2-DMb1	protein_coding	1.21E-02	-3.42	9.45E+00	1.13E-03	2.93E-01	4.42E-01	1.51E+00
H2-Q7	protein_coding	3.67E-03	-3.41	1.40E+02	1.68E-04	2.93E-01	6.52E+00	2.22E+01
Dct	protein_coding	1.90E-03	-3.32	1.19E+01	5.19E-05	3.01E-01	5.44E-01	1.81E+00
Gm43565	lincRNA	3.14E-03	-3.27	9.42E+00	1.33E-04	3.06E-01	4.69E-01	1.53E+00
C7	protein_coding	9.31E-04	-3.25	4.03E+01	1.37E-05	3.07E-01	1.97E+00	6.39E+00
Gm43042	lincRNA	2.18E-02	-3.20	6.52E+00	2.92E-03	3.12E-01	2.78E-01	8.89E-01
Rtn4r	protein_coding	2.27E-04	-3.17	5.15E+01	1.05E-06	3.15E-01	2.45E+00	7.76E+00
Gm4724	protein_coding	2.59E-02	-3.14	1.11E+01	3.83E-03	3.18E-01	4.69E-01	1.47E+00
Prnd	protein_coding	8.59E-04	-3.11	3.72E+01	1.08E-05	3.21E-01	1.65E+00	5.13E+00
Aplnr	protein_coding	1.42E-03	-3.05	1.92E+02	2.99E-05	3.27E-01	9.57E+00	2.92E+01
Retnla	protein_coding	2.76E-03	-3.05	2.40E+01	1.05E-04	3.28E-01	1.33E+00	4.07E+00
Gbp3	protein_coding	1.61E-02	-3.02	2.34E+02	1.81E-03	3.31E-01	1.17E+01	3.53E+01
H2-Q6	protein_coding	3.82E-03	-2.98	1.37E+02	1.83E-04	3.36E-01	7.05E+00	2.10E+01
2310069G16Rik	lincRNA	1.87E-03	-2.92	8.57E+00	4.97E-05	3.42E-01	4.56E-01	1.33E+00
Gbp2	protein_coding	2.58E-02	-2.90	1.43E+02	3.79E-03	3.45E-01	7.38E+00	2.14E+01
AW112010	lincRNA	8.17E-04	-2.80	9.95E+01	9.92E-06	3.57E-01	5.43E+00	1.52E+01
Igsf9	protein_coding	5.55E-03	-2.76	6.82E+00	3.25E-04	3.62E-01	3.66E-01	1.01E+00
Ano5	protein_coding	5.05E-03	-2.73	1.86E+01	2.86E-04	3.66E-01	1.02E+00	2.79E+00
Gbp7	protein_coding	3.94E-02	-2.73	2.21E+02	7.03E-03	3.67E-01	1.20E+01	3.26E+01
Has3	protein_coding	1.90E-03	-2.73	2.00E+01	5.21E-05	3.67E-01	1.01E+00	2.76E+00
Fcgr1	protein_coding	2.01E-03	-2.72	1.03E+01	5.75E-05	3.68E-01	5.73E-01	1.56E+00
Gm7609	transcribed_unprocessed_ pseudogene	1.81E-02	-2.71	1.64E+01	2.19E-03	3.70E-01	9.25E-01	2.50E+00
Gbp5	protein_coding	2.14E-02	-2.70	8.69E+01	2.83E-03	3.70E-01	4.59E+00	1.24E+01
Lamc3	protein_coding	1.60E-02	-2.69	6.64E+00	1.79E-03	3.71E-01	3.65E-01	9.83E-01
Psmb8	protein_coding	5.88E-03	-2.66	9.21E+01	3.59E-04	3.76E-01	5.06E+00	1.35E+01
Adap1	protein_coding	9.08E-04	-2.64	1.70E+01	1.25E-05	3.79E-01	9.88E-01	2.61E+00
Scgb1c1	protein_coding	8.23E-03	-2.63	4.71E+01	6.24E-04	3.80E-01	2.73E+00	7.19E+00
Hr	protein_coding	3.80E-05	-2.63	1.03E+02	4.60E-08	3.80E-01	5.65E+00	1.49E+01

Slc38a1	protein_coding	1.76E-03	-2.58	6.41E+01	4.50E-05	3.88E-01	3.60E+00	9.29E+00
Cdkn1c	protein_coding	9.05E-04	-2.57	6.93E+01	1.21E-05	3.89E-01	3.91E+00	1.00E+01
Arhgap4	protein_coding	1.39E-03	-2.55	1.48E+01	2.82E-05	3.92E-01	8.39E-01	2.14E+00
Scn3b	protein_coding	3.10E-03	-2.53	6.18E+00	1.29E-04	3.96E-01	3.47E-01	8.77E-01
Gm28557	protein_coding	1.36E-02	-2.50	1.36E+01	1.38E-03	4.00E-01	6.92E-01	1.73E+00
Gm11127	protein_coding	2.67E-02	-2.50	7.61E+01	4.01E-03	4.00E-01	4.38E+00	1.09E+01
Irf4	protein_coding	1.68E-02	-2.49	8.73E+00	1.94E-03	4.01E-01	5.29E-01	1.32E+00
Rbm12b1	protein_coding	1.90E-03	-2.49	7.99E+01	5.20E-05	4.01E-01	4.55E+00	1.13E+01
Ifit1	protein_coding	1.63E-02	-2.48	4.77E+01	1.86E-03	4.03E-01	2.52E+00	6.26E+00
H2-Q5	polymorphic_pseudogene	1.43E-02	-2.47	3.65E+01	1.50E-03	4.04E-01	2.12E+00	5.24E+00
Foxo6	protein_coding	8.40E-03	-2.45	6.99E+00	6.48E-04	4.09E-01	3.69E-01	9.03E-01
Herc6	protein_coding	2.45E-03	-2.45	5.42E+01	8.41E-05	4.09E-01	3.15E+00	7.71E+00
Rps6kl1	protein_coding	2.28E-03	-2.42	1.34E+01	7.38E-05	4.13E-01	7.92E-01	1.91E+00
Rgs7	protein_coding	4.18E-02	-2.41	7.45E+00	7.75E-03	4.14E-01	4.61E-01	1.11E+00
Gm44777	antisense	2.55E-02	-2.41	6.17E+00	3.73E-03	4.16E-01	3.46E-01	8.33E-01
Timeless	protein_coding	1.27E-03	-2.40	7.65E+01	2.48E-05	4.16E-01	4.52E+00	1.09E+01
Mgl2	protein_coding	7.28E-03	-2.38	2.03E+01	5.19E-04	4.20E-01	1.30E+00	3.11E+00
Usp18	protein_coding	8.68E-03	-2.38	1.88E+01	6.81E-04	4.21E-01	1.06E+00	2.52E+00
Ifit3	protein_coding	1.15E-02	-2.37	8.88E+01	1.04E-03	4.23E-01	5.04E+00	1.19E+01
Fgd2	protein_coding	2.55E-03	-2.36	1.27E+01	9.11E-05	4.23E-01	7.74E-01	1.83E+00
Fgf6	protein_coding	1.32E-02	-2.35	1.16E+01	1.32E-03	4.25E-01	7.22E-01	1.70E+00
Ccr5	protein_coding	1.99E-04	-2.34	3.23E+01	8.51E-07	4.27E-01	1.89E+00	4.44E+00
Slfn8	protein_coding	4.64E-03	-2.34	1.85E+01	2.53E-04	4.28E-01	1.13E+00	2.65E+00
H2-DMb2	protein_coding	1.74E-02	-2.33	8.46E+00	2.05E-03	4.29E-01	5.34E-01	1.25E+00
B2m	protein_coding	7.28E-03	-2.33	1.42E+03	5.20E-04	4.29E-01	8.71E+01	2.03E+02
Gemin4	protein_coding	4.05E-03	-2.33	1.98E+01	2.03E-04	4.30E-01	1.00E+00	2.33E+00
Helt	protein_coding	5.46E-03	-2.32	7.25E+00	3.17E-04	4.30E-01	4.28E-01	9.95E-01
Acap1	protein_coding	1.91E-02	-2.32	6.02E+00	2.37E-03	4.31E-01	3.68E-01	8.53E-01

Phlda1	protein_coding	1.98E-02	-2.31	7.50E+01	2.52E-03	4.33E-01	4.78E+00	1.10E+01
Ifit3b	protein_coding	4.78E-02	-2.31	2.97E+01	9.40E-03	4.33E-01	1.66E+00	3.83E+00
Bst2	protein_coding	1.26E-02	-2.31	5.92E+01	1.21E-03	4.33E-01	3.56E+00	8.22E+00
Ifi44	protein_coding	4.29E-03	-2.30	2.48E+01	2.24E-04	4.34E-01	1.50E+00	3.46E+00
H2-Q4	protein_coding	7.08E-03	-2.30	3.23E+02	4.93E-04	4.35E-01	1.98E+01	4.56E+01
Cpxm2	protein_coding	9.11E-03	-2.27	2.07E+02	7.20E-04	4.40E-01	1.38E+01	3.15E+01
Fam212b	protein_coding	2.52E-03	-2.27	1.52E+02	8.80E-05	4.41E-01	8.92E+00	2.02E+01
F730043M19Rik	bidirectional_promoter_lncRNA	1.09E-02	-2.26	8.30E+00	9.60E-04	4.42E-01	4.94E-01	1.12E+00
Cys1	protein_coding	1.31E-03	-2.26	2.11E+01	2.56E-05	4.42E-01	1.17E+00	2.65E+00
H2-K1	protein_coding	6.75E-03	-2.26	7.78E+02	4.55E-04	4.42E-01	4.89E+01	1.11E+02
H2-DMa	protein_coding	2.97E-03	-2.23	1.67E+01	1.22E-04	4.48E-01	1.06E+00	2.36E+00
Metrn	protein_coding	4.19E-04	-2.23	7.21E+01	3.32E-06	4.49E-01	4.60E+00	1.02E+01
Ifi209	protein_coding	9.79E-03	-2.22	9.35E+00	8.16E-04	4.50E-01	6.15E-01	1.36E+00
Plau	protein_coding	5.09E-04	-2.20	3.90E+01	4.42E-06	4.54E-01	2.42E+00	5.34E+00
Ccdc88b	protein_coding	1.32E-02	-2.20	1.09E+01	1.30E-03	4.54E-01	6.80E-01	1.50E+00
Ppp1r3b	protein_coding	1.23E-02	-2.19	2.48E+02	1.17E-03	4.56E-01	1.53E+01	3.36E+01
Wisp2	protein_coding	5.72E-03	-2.19	4.68E+01	3.44E-04	4.57E-01	2.91E+00	6.36E+00
Rtp4	protein_coding	2.59E-02	-2.18	3.60E+01	3.83E-03	4.58E-01	2.24E+00	4.90E+00
Rnd1	protein_coding	9.66E-03	-2.18	9.09E+00	7.90E-04	4.58E-01	5.44E-01	1.19E+00
Ier5l	protein_coding	1.64E-02	-2.18E	1.06E+01	1.87E-03	4.59E-01	6.59E-01	1.44E+00
Dctd	protein_coding	3.27E-03	-2.17	5.75E+00	1.41E-04	4.60E-01	3.55E-01	7.72E-01
Hsph1	protein_coding	3.96E-04	-2.17	2.82E+02	2.99E-06	4.60E-01	1.82E+01	3.94E+01
Ip6k3	protein_coding	3.88E-04	-2.17	9.17E+01	2.78E-06	4.61E-01	5.67E+00	1.23E+01
Gins2	protein_coding	2.36E-03	-2.17	6.58E+00	7.85E-05	4.61E-01	3.83E-01	8.30E-01
Pgbd1	protein_coding	1.06E-02	-2.17	1.12E+01	9.27E-04	4.61E-01	6.21E-01	1.35E+00
Nrg1	protein_coding	1.62E-02	-2.17	8.21E+00	1.84E-03	4.62E-01	5.02E-01	1.09E+00
Gm20559	processed_transcript	1.42E-03	-2.16	6.52E+01	2.98E-05	4.62E-01	4.05E+00	8.77E+00
H2-Q10	protein_coding	3.04E-02	-2.14	3.82E+01	4.82E-03	4.66E-01	2.45E+00	5.25E+00

Zfp691	protein_coding	3.42E-03	-2.14	6.17E+01	1.50E-04	4.67E-01	3.64E+00	7.78E+00
Gm45909	TEC	4.23E-02	-2.13	1.06E+01	7.88E-03	4.70E-01	6.55E-01	1.39E+00
Timd4	protein_coding	3.10E-03	-2.13	1.31E+01	1.29E-04	4.70E-01	7.94E-01	1.69E+00
A330009N23Rik	lincRNA	2.95E-02	-2.11	7.68E+00	4.61E-03	4.74E-01	4.71E-01	9.93E-01
Gm29007	lincRNA	3.61E-02	-2.11	6.09E+00	6.16E-03	4.74E-01	4.07E-01	8.58E-01
Gm1305	protein_coding	2.94E-02	-2.11	6.77E+00	4.58E-03	4.74E-01	4.14E-01	8.74E-01
Pcdh12	protein_coding	1.00E-03	-2.10	1.52E+02	1.59E-05	4.75E-01	9.34E+00	1.97E+01
Meig1	protein_coding	7.31E-03	-2.09	1.60E+01	5.23E-04	4.78E-01	1.07E+00	2.23E+00
Nrarp	protein_coding	5.86E-03	-2.09	5.53E+01	3.57E-04	4.79E-01	3.57E+00	7.46E+00
Slfn9	protein_coding	3.41E-02	-2.09	2.30E+01	5.68E-03	4.79E-01	1.50E+00	3.13E+00
Gm10499	transcribed_unprocessed_ pseudogene	1.39E-02	-2.08	7.48E+01	1.44E-03	4.80E-01	4.98E+00	1.04E+01
2610306O10Rik	TEC	9.17E-03	-2.07	8.85E+00	7.27E-04	4.82E-01	5.45E-01	1.13E+00
Aif1	protein_coding	1.87E-02	-2.07	1.06E+01	2.29E-03	4.83E-01	7.11E-01	1.47E+00
Igsf9b	protein_coding	4.06E-03	-2.07	8.00E+01	2.05E-04	4.83E-01	4.97E+00	1.03E+01
Pdzd3	protein_coding	1.49E-03	-2.07	2.94E+01	3.27E-05	4.83E-01	1.96E+00	4.06E+00
Tnfrsf25	protein_coding	3.70E-02	-2.07	1.18E+01	6.43E-03	4.83E-01	7.74E-01	1.60E+00
Gnb3	protein_coding	3.18E-02	-2.07	9.97E+01	5.15E-03	4.84E-01	6.92E+00	1.43E+01
Akap5	protein_coding	7.69E-03	-2.06	4.14E+01	5.63E-04	4.85E-01	2.70E+00	5.57E+00
Rimkla	protein_coding	2.31E-02	-2.06	7.32E+00	3.19E-03	4.86E-01	4.70E-01	9.69E-01
Ret	protein_coding	3.13E-03	-2.05	2.34E+01	1.32E-04	4.87E-01	1.50E+00	3.08E+00
Parp10	protein_coding	5.03E-03	-2.05	1.02E+02	2.83E-04	4.87E-01	6.77E+00	1.39E+01
Zfp658	protein_coding	2.15E-02	-2.05	1.29E+01	2.85E-03	4.88E-01	7.80E-01	1.60E+00
Ptpn7	protein_coding	2.67E-02	-2.05	6.27E+00	4.00E-03	4.88E-01	3.69E-01	7.55E-01
Nr4a1	protein_coding	2.35E-02	-2.05	6.06E+01	3.28E-03	4.89E-01	3.96E+00	8.10E+00
H2-T22	protein_coding	6.97E-03	-2.05	1.34E+02	4.77E-04	4.89E-01	9.01E+00	1.84E+01
Cfap126	protein_coding	1.08E-02	-2.04	6.07E+00	9.44E-04	4.91E-01	4.16E-01	8.46E-01
Ttc39a	protein_coding	2.90E-02	-2.03	9.13E+00	4.49E-03	4.92E-01	5.93E-01	1.20E+00
H2-D1	protein_coding	4.75E-03	-2.03	1.05E+03	2.61E-04	4.92E-01	7.07E+01	1.44E+02

Samd5	protein_coding	1.11E-03	-2.02	3.62E+01	1.97E-05	4.94E-01	2.31E+00	4.67E+00
Spc24	protein_coding	4.07E-03	-2.02	1.68E+01	2.07E-04	4.94E-01	1.07E+00	2.16E+00
Bcl11b	protein_coding	1.15E-02	-2.02	1.33E+01	1.06E-03	4.95E-01	7.37E-01	1.49E+00
Neurl3	protein_coding	2.95E-03	-2.02	3.15E+01	1.19E-04	4.95E-01	2.01E+00	4.06E+00
H2-T24	protein_coding	2.55E-03	-2.02	1.20E+02	9.09E-05	4.96E-01	7.82E+00	1.58E+01
Fam19a3	protein_coding	8.17E-04	-2.01	2.62E+01	9.95E-06	4.98E-01	1.74E+00	3.49E+00
Ifit2	protein_coding	2.20E-02	-2.01	1.44E+02	2.96E-03	4.98E-01	9.43E+00	1.89E+01
Fgf11	protein_coding	2.04E-03	-2.01	1.90E+01	6.12E-05	4.98E-01	1.18E+00	2.36E+00
Cited4	protein_coding	2.22E-02	-2.00	5.42E+01	3.01E-03	4.99E-01	3.53E+00	7.06E+00
Oasl2	protein_coding	7.49E-03	-2.00	7.88E+01	5.41E-04	5.00E-01	5.14E+00	1.03E+01
Gsta1	protein_coding	8.54E-03	2.01	1.09E+01	6.62E-04	2.01E+00	1.50E+00	7.49E-01
Zc3h6	protein_coding	1.01E-02	2.01	6.67E+01	8.55E-04	2.01E+00	9.04E+00	4.50E+00
Tcf7	protein_coding	2.02E-02	2.02	9.90E+00	2.61E-03	2.02E+00	1.42E+00	7.05E-01
Ctgf	protein_coding	4.27E-03	2.02	4.28E+02	2.22E-04	2.02E+00	6.29E+01	3.11E+01
Igf2	protein_coding	8.84E-04	2.02	1.59E+02	1.16E-05	2.02E+00	2.16E+01	1.07E+01
Fzd8	protein_coding	8.79E-04	2.02	3.92E+01	1.15E-05	2.02E+00	5.56E+00	2.75E+00
Lama5	protein_coding	8.79E-04	2.02	1.55E+03	1.15E-05	2.02E+00	2.06E+02	1.02E+02
Dixdc1	protein_coding	7.69E-03	2.02	7.49E+01	5.63E-04	2.02E+00	1.05E+01	5.19E+00
Igsf23	protein_coding	4.00E-02	2.03	5.73E+00	7.20E-03	2.03E+00	7.68E-01	3.79E-01
Rgs17	protein_coding	8.39E-03	2.04	1.25E+01	6.46E-04	2.04E+00	1.77E+00	8.71E-01
Thbs1	protein_coding	2.47E-02	2.04	6.30E+01	3.55E-03	2.04E+00	8.27E+00	4.06E+00
Acsm5	protein_coding	1.08E-03	2.04	7.69E+01	1.82E-05	2.04E+00	1.05E+01	5.16E+00
Gm45357	lincRNA	4.26E-03	2.04	1.18E+01	2.21E-04	2.04E+00	1.55E+00	7.60E-01
Asb4	protein_coding	9.78E-04	2.05	4.50E+01	1.53E-05	2.05E+00	6.10E+00	2.98E+00
Pbld1	protein_coding	4.11E-03	2.05	1.08E+01	2.10E-04	2.05E+00	1.58E+00	7.71E-01
Tox	protein_coding	5.86E-03	2.06	7.79E+00	3.57E-04	2.06E+00	9.61E-01	4.68E-01
RP23-438P19.7	sense_intronic	1.99E-02	2.07	1.64E+01	2.55E-03	2.07E+00	2.59E+00	1.25E+00
Ahsg	protein_coding	1.11E-02	2.07	1.76E+01	9.91E-04	2.07E+00	2.44E+00	1.18E+00

Acot3	protein_coding	3.81E-03	2.07	1.40E+01	1.81E-04	2.07E+00	1.90E+00	9.15E-01
Gm12100	processed_transcript	1.11E-02	2.07	9.06E+00	9.89E-04	2.07E+00	1.11E+00	5.33E-01
Cited2	protein_coding	8.17E-04	2.09	1.95E+02	1.00E-05	2.09E+00	2.60E+01	1.24E+01
Mmp3	protein_coding	1.52E-02	2.10	9.32E+00	1.67E-03	2.10E+00	1.29E+00	6.17E-01
Fam107a	protein_coding	1.34E-03	2.11	9.48E+01	2.63E-05	2.11E+00	1.49E+01	7.08E+00
Cpa3	protein_coding	1.84E-02	2.13	1.24E+01	2.25E-03	2.13E+00	1.71E+00	8.04E-01
Ehhadh	protein_coding	1.98E-04	2.14	1.63E+02	7.63E-07	2.14E+00	2.28E+01	1.07E+01
Lipe	protein_coding	5.43E-05	2.14	3.75E+02	8.91E-08	2.14E+00	5.31E+01	2.48E+01
Inhbb	protein_coding	7.32E-04	2.15	2.11E+01	8.50E-06	2.15E+00	2.91E+00	1.35E+00
Fam122b	protein_coding	9.08E-04	2.16	6.58E+01	1.25E-05	2.16E+00	8.61E+00	3.99E+00
Fbp2	protein_coding	4.98E-04	2.16	2.24E+02	4.26E-06	2.16E+00	3.23E+01	1.49E+01
Enc1	protein_coding	3.88E-04	2.17	9.74E+01	2.75E-06	2.17E+00	1.40E+01	6.46E+00
Gabrr2	protein_coding	1.24E-03	2.17	1.54E+01	2.37E-05	2.17E+00	2.03E+00	9.38E-01
Rasef	protein_coding	5.42E-03	2.20	1.13E+01	3.13E-04	2.20E+00	1.57E+00	7.13E-01
Gm45716	protein_coding	9.32E-05	2.22	6.42E+01	2.11E-07	2.22E+00	9.03E+00	4.06E+00
Ncam1	protein_coding	5.46E-03	2.22	3.30E+01	3.17E-04	2.22E+00	4.31E+00	1.94E+00
Acot2	protein_coding	9.32E-05	2.23	7.03E+02	2.13E-07	2.23E+00	1.04E+02	4.65E+01
Sbk3	protein_coding	5.77E-04	2.24	2.74E+01	5.47E-06	2.24E+00	3.81E+00	1.70E+00
Aldob	protein_coding	1.17E-02	2.24	4.69E+01	1.07E-03	2.24E+00	6.38E+00	2.84E+00
Kif6	protein_coding	2.22E-03	2.25	1.21E+01	7.07E-05	2.25E+00	1.85E+00	8.24E-01
Ubxn10	protein_coding	7.38E-03	2.26	1.42E+01	5.31E-04	2.26E+00	2.06E+00	9.13E-01
Txnip	protein_coding	2.64E-03	2.26	5.19E+03	9.73E-05	2.26E+00	8.11E+02	3.59E+02
Pirt	protein_coding	1.60E-02	2.26	8.91E+01	1.80E-03	2.26E+00	1.13E+01	4.98E+00
Tpsb2	protein_coding	1.21E-02	2.27	7.39E+00	1.12E-03	2.27E+00	1.13E+00	4.95E-01
Cldn15	protein_coding	2.95E-03	2.28	3.03E+01	1.19E-04	2.28E+00	4.37E+00	1.92E+00
Gm12295	lincRNA	3.09E-04	2.30	7.29E+01	1.84E-06	2.30E+00	1.06E+01	4.59E+00
Vsig4	protein_coding	6.38E-03	2.33	1.88E+01	4.06E-04	2.33E+00	2.65E+00	1.14E+00
Adra1b	protein_coding	1.93E-04	2.35	1.84E+02	7.31E-07	2.35E+00	2.57E+01	1.10E+01

Lcn2	protein_coding	9.23E-04	2.36	1.78E+01	1.32E-05	2.36E+00	2.14E+00	9.07E-01
Fkbp5	protein_coding	1.76E-04	2.38	2.31E+02	5.78E-07	2.38E+00	3.35E+01	1.41E+01
Gpr160	protein_coding	6.13E-04	2.41	2.65E+01	5.94E-06	2.41E+00	4.03E+00	1.67E+00
Cacng6	protein_coding	2.45E-03	2.42	8.67E+00	8.37E-05	2.42E+00	1.16E+00	4.80E-01
Gm12519	lincRNA	6.49E-03	2.42	8.50E+00	4.26E-04	2.42E+00	1.38E+00	5.70E-01
Pcdhga8	protein_coding	3.49E-03	2.42	7.64E+00	1.56E-04	2.42E+00	1.13E+00	4.65E-01
Col5a3	protein_coding	8.40E-03	2.44	9.27E+01	6.49E-04	2.44E+00	1.41E+01	5.78E+00
H19	lincRNA	1.96E-03	2.46	1.39E+02	5.49E-05	2.46E+00	2.02E+01	8.18E+00
Tcf23	protein_coding	5.85E-03	2.47	2.62E+01	3.54E-04	2.47E+00	3.88E+00	1.57E+00
Rasd1	protein_coding	2.01E-03	2.48	2.24E+01	5.84E-05	2.48E+00	3.28E+00	1.33E+00
Rgs2	protein_coding	1.07E-03	2.49	1.71E+02	1.79E-05	2.49E+00	2.68E+01	1.08E+01
Btnl9	protein_coding	4.29E-05	2.49	6.78E+02	6.24E-08	2.49E+00	1.05E+02	4.23E+01
Hrct1	protein_coding	9.65E-04	2.50	3.56E+01	1.48E-05	2.50E+00	5.68E+00	2.27E+00
Gm17251	processed_transcript	7.21E-04	2.50	2.18E+01	8.22E-06	2.50E+00	3.46E+00	1.38E+00
Pdzrn4	protein_coding	6.39E-04	2.50	1.16E+01	6.60E-06	2.50E+00	1.51E+00	6.03E-01
Cdo1	protein_coding	2.39E-02	2.54	9.57E+00	3.36E-03	2.54E+00	1.38E+00	5.44E-01
Clca3a1	protein_coding	4.75E-03	2.55	1.04E+01	2.62E-04	2.55E+00	1.70E+00	6.67E-01
Ddit4	protein_coding	6.50E-03	2.55	3.19E+02	4.28E-04	2.55E+00	4.94E+01	1.94E+01
Snai3	protein_coding	3.67E-03	2.57	5.93E+01	1.70E-04	2.57E+00	7.72E+00	3.00E+00
Adamts12	protein_coding	6.91E-04	2.60	1.27E+01	7.65E-06	2.60E+00	1.98E+00	7.63E-01
Cenpf	protein_coding	1.34E-02	2.60	1.20E+02	1.35E-03	2.60E+00	1.67E+01	6.40E+00
Mthfd2	protein_coding	2.34E-03	2.62	1.90E+01	7.74E-05	2.62E+00	3.15E+00	1.20E+00
Gm14964	antisense	2.88E-03	2.62	8.98E+00	1.13E-04	2.62E+00	1.38E+00	5.25E-01
Cyp1b1	protein_coding	2.89E-03	2.63	6.09E+01	1.14E-04	2.63E+00	1.01E+01	3.85E+00
Gm38218	TEC	1.35E-03	2.66	9.35E+00	2.71E-05	2.66E+00	1.39E+00	5.23E-01
Lox	protein_coding	7.28E-04	2.68	3.52E+01	8.36E-06	2.68E+00	5.29E+00	1.97E+00
Gm16316	antisense	4.06E-03	2.69	1.05E+01	2.05E-04	2.69E+00	1.60E+00	5.93E-01
Spon2	protein_coding	3.66E-02	2.71	1.31E+01	6.31E-03	2.71E+00	2.12E+00	7.85E-01

Gm26703	antisense	1.56E-03	2.73	1.48E+01	3.48E-05	2.73E+00	2.30E+00	8.42E-01
Pth1r	protein_coding	1.35E-04	2.80	3.15E+01	4.14E-07	2.80E+00	5.08E+00	1.81E+00
Lgals4	protein_coding	1.40E-05	2.85	6.00E+02	2.77E-09	2.85E+00	9.48E+01	3.33E+01
Gm31659	lincRNA	3.13E-03	2.87	1.68E+01	1.32E-04	2.87E+00	2.30E+00	8.01E-01
Agt	protein_coding	1.89E-03	2.90	3.41E+01	5.09E-05	2.90E+00	4.78E+00	1.65E+00
Cox6b2	protein_coding	9.91E-04	2.91	1.73E+01	1.55E-05	2.91E+00	2.77E+00	9.53E-01
Myc	protein_coding	1.30E-02	2.92	2.20E+01	1.28E-03	2.92E+00	3.71E+00	1.27E+00
Vipr2	protein_coding	2.88E-03	2.94	8.75E+00	1.13E-04	2.94E+00	1.44E+00	4.89E-01
Cyp1a1	protein_coding	1.55E-02	2.96	1.47E+01	1.72E-03	2.96E+00	2.43E+00	8.23E-01
Clec12b	protein_coding	6.72E-03	3.00	5.74E+00	4.50E-04	3.00E+00	9.33E-01	3.11E-01
Vwa3a	protein_coding	1.40E-05	3.01	9.74E+01	2.35E-09	3.01E+00	1.72E+01	5.73E+00
Spock2	protein_coding	2.04E-05	3.01	1.48E+02	8.87E-09	3.01E+00	2.17E+01	7.20E+00
Edn3	protein_coding	6.13E-03	3.01	4.72E+01	3.82E-04	3.01E+00	6.89E+00	2.29E+00
Arrdc2	protein_coding	1.97E-02	3.03	4.94E+01	2.50E-03	3.03E+00	8.45E+00	2.79E+00
Slc41a3	protein_coding	2.01E-03	3.07	2.03E+02	5.80E-05	3.07E+00	3.11E+01	1.02E+01
Gm45805	lincRNA	3.12E-03	3.07	6.99E+00	1.31E-04	3.07E+00	1.15E+00	3.75E-01
Gm3776	protein_coding	2.73E-02	3.09	5.53E+00	4.15E-03	3.09E+00	9.27E-01	3.00E-01
Arntl	protein_coding	3.32E-02	3.12	9.14E+00	5.45E-03	3.12E+00	1.54E+00	4.93E-01
Lyz1	protein_coding	4.32E-03	3.13	6.70E+00	2.26E-04	3.13E+00	1.06E+00	3.39E-01
Aqp4	protein_coding	9.21E-04	3.15	5.42E+01	1.31E-05	3.15E+00	7.45E+00	2.37E+00
Gm43204	lincRNA	9.61E-04	3.18	8.79E+00	1.43E-05	3.18E+00	1.55E+00	4.88E-01
Ucp3	protein_coding	8.73E-04	3.18	8.01E+02	1.12E-05	3.18E+00	1.31E+02	4.11E+01
Rmst	lincRNA	2.51E-03	3.27	8.75E+00	8.75E-05	3.27E+00	1.43E+00	4.38E-01
Abca4	protein_coding	7.65E-05	3.32	4.84E+01	1.44E-07	3.32E+00	7.10E+00	2.14E+00
Gm10603	antisense	1.11E-03	3.34	1.40E+01	1.98E-05	3.34E+00	2.26E+00	6.78E-01
8430408G22Rik	protein_coding	2.75E-05	3.40	3.86E+02	2.94E-08	3.40E+00	6.36E+01	1.87E+01
Kcne1	protein_coding	2.01E-03	3.40	1.00E+02	5.88E-05	3.40E+00	1.48E+01	4.34E+00
Cdkn1a	protein_coding	2.36E-03	3.44	9.82E+01	7.86E-05	3.44E+00	1.73E+01	5.03E+00

Gm10032	protein_coding	2.15E-03	3.48	7.09E+00	6.65E-05	3.48E+00	1.15E+00	3.29E-01
Abhd1	processed_transcript	3.40E-04	3.50	3.53E+01	2.13E-06	3.50E+00	6.26E+00	1.79E+00
Chrna2	protein_coding	1.58E-03	3.67	4.57E+01	3.70E-05	3.67E+00	8.92E+00	2.43E+00
Scd4	protein_coding	6.91E-04	3.68	2.47E+02	7.56E-06	3.68E+00	4.35E+01	1.18E+01
Sfrp5	protein_coding	2.40E-03	3.72	9.94E+00	8.12E-05	3.72E+00	1.52E+00	4.09E-01
Scube1	protein_coding	4.29E-05	3.76	5.32E+01	6.42E-08	3.76E+00	8.34E+00	2.22E+00
Lrrc52	protein_coding	1.01E-03	3.82	6.26E+00	1.62E-05	3.82E+00	1.10E+00	2.87E-01
Hif3a	protein_coding	3.56E-04	3.97	2.79E+01	2.35E-06	3.97E+00	4.94E+00	1.24E+00
Fam46b	protein_coding	6.91E-04	4.11	8.74E+00	7.50E-06	4.11E+00	1.47E+00	3.58E-01
Klk1b26	protein_coding	1.54E-03	4.18	1.36E+01	3.41E-05	4.18E+00	1.92E+00	4.60E-01
Acot1	protein_coding	2.04E-05	4.28	1.71E+02	1.02E-08	4.28E+00	3.11E+01	7.27E+00
Snhg11	protein_coding	3.61E-02	4.51	1.66E+01	6.16E-03	4.51E+00	2.20E+00	4.87E-01
AI593442	protein_coding	2.21E-03	4.69	2.37E+01	7.02E-05	4.69E+00	3.76E+00	8.01E-01
Pdk4	protein_coding	2.55E-05	6.26	9.97E+03	1.45E-08	6.26E+00	2.07E+03	3.31E+02
Cyp2b10	protein_coding	1.99E-04	6.31	9.99E+00	8.37E-07	6.31E+00	2.00E+00	3.17E-01
Kcnk1	protein_coding	2.09E-03	6.97	7.14E+00	6.33E-05	6.97E+00	1.16E+00	1.66E-01
Sdk2	protein_coding	2.62E-05	7.37	1.91E+01	1.87E-08	7.37E+00	4.16E+00	5.65E-01
Hmgcs2	protein_coding	1.40E-05	7.37	2.84E+02	3.43E-09	7.37E+00	6.28E+01	8.52E+00
Angptl4	protein_coding	9.57E-04	9.59	1.42	1.42E-05	9.59E+00	3.14E+01	3.28E+00

Supplementary Table S7: Genes differentially expressed in hearts from *LAV-BPIFB4*- and vehicle-treated diabetic mice at 1.5 FC.

GENE ID	GENE BIOTYPE	FDR STEP UP	FOLD CHANGE (*LAV vs.* V)	TOTAL COUNTS	*p*-VALUE	RATIO	LS MEAN *LAV*	LS MEAN V
2810474O19Rik	protein_coding	0.04	1.60	232.08	0.00	1.60	29.12	18.23
Acot1	protein_coding	0.03	1.93	170.68	0.00	1.93	31.09	16.11
Bcl2l1	protein_coding	0.00	1.81	240.89	0.00	1.81	34.67	19.18
Btnl9	protein_coding	0.03	1.63	677.76	0.00	1.63	105.11	64.46
Fam107a	protein_coding	0.03	2.05	94.78	0.00	2.05	14.90	7.26
Foxo6os	antisense	0.05	-2.06	40.10	0.00	0.49	1.44	2.96
Gemin4	protein_coding	0.04	-2.50	19.85	0.00	0.40	1.00	2.50
Gm15013	protein_coding	0.05	-1.90	5.46	0.00	0.53	0.34	0.65
Hmgcs2	protein_coding	0.01	3.04	284.34	0.00	3.04	62.80	20.62
Ky	protein_coding	0.02	-1.60	146.50	0.00	0.63	9.66	15.44
Lgals4	protein_coding	0.03	1.56	599.98	0.00	1.56	94.76	60.88
Map1a	protein_coding	0.03	-1.60	61.97	0.00	0.62	4.70	7.53
Map3k6	protein_coding	0.03	1.87	73.50	0.00	1.87	10.69	5.73
Pdk4	protein_coding	0.03	2.57	9970.50	0.00	2.57	2073.76	808.25
Rbp7	protein_coding	0.01	1.79	85.30	0.00	1.79	12.41	6.93
Rgcc	protein_coding	0.04	1.69	112.61	0.00	1.69	15.43	9.11
Sdk2	protein_coding	0.03	2.85	19.14	0.00	2.85	4.16	1.46
Timp4	protein_coding	0.04	1.57	395.05	0.00	1.57	49.29	31.42
Tlr1	protein_coding	0.03	-2.01	8.09	0.00	0.50	0.36	0.71
Vwa3a	protein_coding	0.00	2.26	97.41	0.00	2.26	17.22	7.61

Supplementary Table S8: Genes differentially expressed in hearts from *LAV-BPIFB4*- and vehicle-treated diabetic mice regardless the FC.

GENE ID	GENE BIOTYPE	FDR STEP UP	FOLD CHANGE (*LAV vs.* V)	TOTAL COUNTS	*p*-VALUE	RATIO	LS MEAN *LAV*	LS MEAN V
Gemin4	protein_coding	4.03E-02	-2.50	1.98E+01	1.44E-04	4.00E-01	1.00E+00	2.50E+00
Foxo6os	antisense	4.66E-02	-2.06	4.01E+01	2.09E-04	4.86E-01	1.44E+00	2.96E+00
Tlr1	protein_coding	3.28E-02	-2.01	8.09E+00	7.94E-05	4.98E-01	3.55E-01	7.13E-01
Gm15013	protein_coding	4.59E-02	-1.90	5.46E+00	1.98E-04	5.27E-01	3.45E-01	6.54E-01
Map1a	protein_coding	2.52E-02	-1.60	6.20E+01	2.98E-05	6.24E-01	4.70E+00	7.53E+00
Ky	protein_coding	2.46E-02	-1.60	1.46E+02	1.93E-05	6.25E-01	9.66E+00	1.54E+01
Vcam1	protein_coding	3.93E-02	-1.43	1.15E+02	1.26E-04	7.00E-01	8.42E+00	1.20E+01
Phyhip	protein_coding	4.03E-02	-1.40	1.05E+02	1.47E-04	7.12E-01	8.29E+00	1.16E+01
Six5	protein_coding	3.11E-02	-1.29	5.44E+01	6.65E-05	7.73E-01	4.58E+00	5.93E+00
Tmem218	protein_coding	4.34E-02	-1.28	6.55E+01	1.70E-04	7.81E-01	5.50E+00	7.04E+00
Sdpr	protein_coding	4.46E-02	-1.22	1.28E+03	1.85E-04	8.19E-01	1.11E+02	1.35E+02
Eno3	protein_coding	2.52E-02	-1.22	4.50E+03	2.21E-05	8.23E-01	3.91E+02	4.76E+02
Tuba4a	protein_coding	2.52E-02	-1.17	3.63E+03	3.41E-05	8.54E-01	3.18E+02	3.73E+02
Mrpl45	protein_coding	4.66E-02	-1.12	4.28E+02	2.13E-04	8.94E-01	4.21E+01	4.71E+01
Atp5sl	protein_coding	3.71E-02	-1.11	2.62E+02	1.05E-04	9.01E-01	2.56E+01	2.84E+01
Utp3	protein_coding	4.46E-02	1.12	1.75E+02	1.82E-04	1.12E+00	1.87E+01	1.66E+01
Etfdh	protein_coding	3.71E-02	1.18	5.48E+03	1.09E-04	1.18E+00	6.63E+02	5.61E+02
Scarb2	protein_coding	3.11E-02	1.20	1.55E+03	5.69E-05	1.20E+00	1.82E+02	1.52E+02
Dhrs4	protein_coding	2.34E-02	1.22	4.54E+02	1.67E-05	1.22E+00	5.37E+01	4.42E+01
Vldlr	protein_coding	4.59E-02	1.22	4.04E+03	2.00E-04	1.22E+00	4.74E+02	3.90E+02
Gm13910	processed_pseudogene	4.34E-02	1.22	1.36E+04	1.70E-04	1.22E+00	1.59E+03	1.30E+03
Cat	protein_coding	4.03E-02	1.23	2.13E+03	1.44E-04	1.23E+00	2.57E+02	2.09E+02
Rlf	protein_coding	2.12E-02	1.24	2.08E+02	1.36E-05	1.24E+00	2.42E+01	1.94E+01
Pdk1	protein_coding	3.11E-02	1.25	1.72E+03	5.28E-05	1.25E+00	2.03E+02	1.63E+02
Hadha	protein_coding	2.95E-02	1.25	1.25E+04	4.62E-05	1.25E+00	1.57E+03	1.25E+03
Ubxn7	protein_coding	4.34E-02	1.26	2.50E+02	1.73E-04	1.26E+00	2.95E+01	2.35E+01

Grcc10	protein_coding	3.43E-02	1.26	4.18E+02	8.89E-05	1.26E+00	4.97E+01	3.94E+01
Clpx	protein_coding	3.73E-02	1.27	8.39E+02	1.12E-04	1.27E+00	1.02E+02	8.04E+01
Pex11a	protein_coding	4.34E-02	1.28	2.00E+02	1.72E-04	1.28E+00	2.50E+01	1.95E+01
Cd36	protein_coding	3.13E-02	1.29	1.41E+04	7.20E-05	1.29E+00	1.77E+03	1.38E+03
Adgrf5	protein_coding	3.13E-02	1.30	2.22E+03	7.02E-05	1.30E+00	2.58E+02	1.99E+02
Pdzrn3	protein_coding	4.34E-02	1.30	2.53E+02	1.73E-04	1.30E+00	3.24E+01	2.49E+01
Arl15	protein_coding	2.12E-02	1.30	6.95E+01	9.29E-06	1.30E+00	7.71E+00	5.91E+00
Plin5	protein_coding	4.03E-02	1.31	9.01E+02	1.37E-04	1.31E+00	1.20E+02	9.18E+01
Vwa8	protein_coding	2.12E-02	1.32	2.55E+03	1.17E-05	1.32E+00	3.22E+02	2.43E+02
Dbi	protein_coding	4.03E-02	1.33	6.15E+02	1.41E-04	1.33E+00	8.31E+01	6.26E+01
Ech1	protein_coding	3.11E-02	1.34	9.07E+03	6.43E-05	1.34E+00	1.25E+03	9.33E+02
Pkp4	protein_coding	2.12E-02	1.34	8.24E+02	1.22E-05	1.34E+00	1.05E+02	7.85E+01
Decr1	protein_coding	2.93E-02	1.36	4.25E+03	4.38E-05	1.36E+00	5.73E+02	4.21E+02
Gpihbp1	protein_coding	2.63E-02	1.37	4.93E+02	3.76E-05	1.37E+00	6.19E+01	4.50E+01
Snhg5	processed_ transcript	3.11E-02	1.39	2.93E+01	6.06E-05	1.39E+00	3.58E+00	2.57E+00
Ifrd1	protein_coding	4.46E-02	1.40	2.04E+02	1.87E-04	1.40E+00	2.41E+01	1.72E+01
Acer2	protein_coding	3.13E-02	1.41	2.62E+02	7.36E-05	1.41E+00	3.28E+01	2.32E+01
Grasp	protein_coding	3.43E-02	1.44	3.01E+01	9.05E-05	1.44E+00	3.40E+00	2.36E+00
Sgk1	protein_coding	1.54E-02	1.44	4.92E+02	5.49E-06	1.44E+00	6.91E+01	4.80E+01
Snhg1	processed_trans cript	3.93E-02	1.44	6.29E+01	1.26E-04	1.44E+00	7.20E+00	4.99E+00
Rassf4	protein_coding	2.52E-02	1.47E	1.60E+02	3.25E-05	1.47E+00	2.14E+01	1.45E+01
Fabp4	protein_coding	2.52E-02	1.48	4.02E+03	2.85E-05	1.48E+00	5.26E+02	3.57E+02
Errfi1	protein_coding	4.63E-02	1.49	1.38E+02	2.04E-04	1.49E+00	1.80E+01	1.21E+01
Aqp7	protein_coding	3.11E-02	1.50	1.34E+02	6.32E-05	1.50E+00	1.81E+01	1.21E+01
Lgals4	protein_coding	3.11E-02	1.56	6.00E+02	6.16E-05	1.56E+00	9.48E+01	6.09E+01
Timp4	protein_coding	3.92E-02	1.57	3.95E+02	1.20E-04	1.57E+00	4.93E+01	3.14E+01

2810474O19Rik	protein_coding	3.71E-02	1.60	2.32E+02	1.07E-04	1.60E+00	2.91E+01	1.82E+01
Btnl9	protein_coding	3.11E-02	1.63	6.78E+02	6.63E-05	1.63E+00	1.05E+02	6.45E+01
Rgcc	protein_coding	4.03E-02	1.69	1.13E+02	1.33E-04	1.69E+00	1.54E+01	9.11E+00
Rbp7	protein_coding	1.43E-02	1.79	8.53E+01	4.00E-06	1.79E+00	1.24E+01	6.93E+00
Bcl2l1	protein_coding	1.81E-03	1.81	2.41E+02	2.58E-07	1.81E+00	3.47E+01	1.92E+01
Map3k6	protein_coding	2.52E-02	1.87	7.35E+01	3.20E-05	1.87E+00	1.07E+01	5.73E+00
Acot1	protein_coding	3.45E-02	1.93	1.71E+02	9.34E-05	1.93E+00	3.11E+01	1.61E+01
Fam107a	protein_coding	3.43E-02	2.05	9.48E+01	8.65E-05	2.05E+00	1.49E+01	7.26E+00
Vwa3a	protein_coding	1.75E-03	2.26	9.74E+01	1.24E-07	2.26E+00	1.72E+01	7.61E+00
Pdk4	protein_coding	2.52E-02	2.57	9.97E+03	3.13E-05	2.57E+00	2.07E+03	8.08E+02
Sdk2	protein_coding	2.52E-02	2.85	1.91E+01	3.33E-05	2.85E+00	4.16E+00	1.46E+00
Hmgcs2	protein_coding	1.43E-02	3.04	2.84E+02	4.06E-06	3.04E+00	6.28E+01	2.06E+01

Supplementary Table S9: qPCR validation of the transcriptome sequencing

Gene	Seq	PCR	Full name	Entrez Gene Summary
Gemin4	-2.50	1.66	Gem Nuclear Organelle Associated Protein 4	The product of this gene is part of a large complex localised to the cytoplasm, nucleoli, and to discrete nuclear bodies called Gemini bodies (gems). The complex functions in spliceosomal snRNP assembly in the cytoplasm, and regenerates spliceosomes required for pre-mRNA splicing in the nucleus. The encoded protein directly interacts with a DEAD box protein and several spliceosome core proteins. Alternatively-spliced transcript variants have been described, but their biological validity has not been determined
Foxo6	-2.06	-1.10	Forkhead Box O6	GO annotations related to this gene include transcription factor activity and sequence-specific DNA binding. An important paralog of this gene is FOXO3.
Tlr1	-2.01	-1.26	Toll Like Receptor 1	Participates in the innate immune response to microbial agents. Specifically recognises diacylated and triacylated lipopeptides. Cooperates with TLR2 to mediate the innate immune response to bacterial lipoproteins or lipopeptides (PubMed:21078852). Forms the activation cluster TLR2:TLR1:CD14 in response to triacylated lipopeptides, this cluster triggers signalling from the cell surface and subsequently is targeted to the Golgi in a lipid-raft dependent pathway (PubMed:16880211). Acts via MYD88 and TRAF6, leading to NF-kappa-B activation, cytokine secretion and the inflammatory response.
Ky	-1.60	1.42	Kyphoscoliosis Peptidase	The protein is involved in the function, maturation and stabilisation of the neuromuscular junction and may be required for normal muscle growth.
Timp4	1.57	2.14	TIMP Metallopeptidase Inhibitor 4	The proteins encoded by this gene family are inhibitors of the matrix metalloproteinases, a group of peptidases involved in degradation of the extracellular matrix. The secreted, netrin domain-containing protein encoded by this gene is involved in regulation of platelet aggregation and recruitment and may play a role in hormonal regulation and endometrial tissue remodelling.
Bcl2l1	1.81	1.91	BCL2 Like 1	The proteins encoded by this gene are located at the outer mitochondrial membrane and have been shown to regulate outer mitochondrial membrane channel (VDAC) opening. VDAC regulates mitochondrial membrane potential, and thus controls the production of reactive oxygen species and release of cytochrome C by mitochondria, both of which are the potent inducers of cell apoptosis.
Map3k6	1.87	1.93	Mitogen-Activated Protein Kinase Kinase Kinase 6	The encoded kinase participates in the regulation of vascular endothelial growth factor (VEGF) expression.
Acot1	1.93	1.75	Acyl-CoA Thioesterase 1	Acyl-CoA thioesterases are a group of enzymes that catalyse the hydrolysis of acyl-CoAs to the free fatty acid and coenzyme A (CoASH), providing the potential to regulate intracellular levels of acyl-CoAs, free fatty acids and CoASH.

Fam107a	2.05	3.36	Family With Sequence Similarity 107 Member A	When transfected into cell lines in which it is not expressed, suppresses cell growth. May play a role in tumour development.
Vwa3a	2.26	1.92	Von Willebrand Factor A Domain Containing 3A	Beyond its role in haemostasis, von Willebrand factor is an emerging mediator of vascular inflammation. VWF and its regulator, ADAMTS13, are implicated in mechanisms that underlie vascular inflammation and immunothrombosis, like leukocyte rolling, adhesion and extravasation and/or vascular permeability and/or ischaemia/reperfusion injury and/or complement activation.
Pdk4	2.57	2.02	Pyruvate Dehydrogenase Kinase 4	The encoded protein plays a key role in regulation of glucose and fatty acid metabolism and homeostasis via phosphorylation of the pyruvate dehydrogenase subunits PDHA1 and PDHA2. This inhibits pyruvate dehydrogenase activity, and thereby regulates metabolite flux through the tricarboxylic acid cycle, down-regulates aerobic respiration and inhibits the formation of acetyl-coenzyme A from pyruvate. Inhibition of pyruvate dehydrogenase decreases glucose utilisation and increases fat metabolism in response to prolonged fasting and starvation. Plays an important role in maintaining normal blood glucose levels under starvation and is involved in the insulin signalling cascade. Via its regulation of pyruvate dehydrogenase activity, plays an important role in maintaining normal blood pH and in preventing the accumulation of ketone bodies under starvation. In the fed state, mediates cellular responses to glucose levels and to a high-fat diet. Regulates both fatty acid oxidation and de novo fatty acid biosynthesis. Plays a role in the generation of reactive oxygen species. Protects detached epithelial cells against anoikis.
Hmgcs2	3.04	2.61	3-Hydroxy-3-Methylglutaryl-CoA Synthase 2	The encoded protein belongs to the HMG-CoA synthase family. It is a mitochondrial enzyme that catalyses the first reaction of ketogenesis, a metabolic pathway that provides lipid-derived energy for various organs during times of carbohydrate deprivation, such as fasting.

The ratio of DE genes assessed by sequencing and qPCR is shown in the respective columns. Information derived from search in https://www.genecards.org/

Supplementary Table S10: Focused transcriptomic analysis identifies components of the mitochondrial fatty acid β-oxidation pathway as targets of *LAV-BPIFB4* therapy.

GENE	PROTEIN NAME	FUNCTION
Acot1	Acyl-CoA thioesterase 1	Catalyses the conversion of acyl-CoA's to free fatty acids and CoA.
AtpB	ATP synthase subunit beta	Production of ATP.
Cs	Citrate Synthase	Converts acetyl-CoA to citrate as part of the mitochondrial TCA cycle
Cpt1b	Carnitine palmitoyltransferase 1B	Transport of long-chain fatty acyl-CoA's from the cytosol into the mitochondria for fatty acid β-oxidation.
Drp1	Dynamin 1 Like	Functions in mitochondrial and peroxisomal division
Hmgcs2	3-Hydroxy-3-Methylglutaryl-CoA Synthase 2	Catalyses the condensation of acetyl-CoA with acetoacetyl-CoA to form HMG-CoA during ketone biosynthesis.
Mcad	Acyl-CoA dehydrogenase (medium chain)	Catalyses the oxidation of medium chain fatty acids.
Mfn2	Mitofusin 2	Involved in mitochondrial fusion, it contributes to the maintenance and operation of the mitochondrial network
PDK4	Pyruvate Dehydrogenase Kinase 4	Regulation of glucose metabolism via inhibition of the mitochondrial pyruvate dehydrogenase complex, thereby reducing mitochondrial pyruvate entry.
Ppargc1α	PPARG Coactivator 1 Alpha	Transcriptional coactivator for steroid receptors and nuclear receptors. Coordinates the expression of genes involved in glucose and fatty acid metabolism.
Tfam	Mitochondrial transcription factor A	Transcriptional regulator of mitochondrial biogenesis.

Tomm20	Translocase of outer mitochondrial membrane 20	Central component of the receptor complex responsible for the recognition and translocation of cytosolically synthesised mitochondrial pre-proteins.
Ucp3	Uncoupling Protein 3	Possible uncoupling of oxidative phosphorylation from ATP synthesis. Transporter of fatty acids between the mitochondrial and cytosol. This gene's protein product is postulated to protect mitochondria against lipid-induced oxidative stress. Expression levels of this gene increase when fatty acid supplies to mitochondria exceed their oxidation capacity and the protein enables the export of fatty acids from mitochondria.
